# Air embolism as a rare complication of lung biopsy: A case report

**DOI:** 10.1016/j.radcr.2024.01.027

**Published:** 2024-01-27

**Authors:** Federica Ricciardella, Gianluca Mannetta, Valentina Caruso, Giulio Cocco, Cesare Mantini, Eleonora Piccirilli, Massimo Caulo, Andrea Delli Pizzi

**Affiliations:** a Department of Radiology, SS. Annunziata Hospital, “G. d'Annunzio” University, Chieti, Via dei Vestini, Chieti, Italy; b Department of Neuroscience, Imaging and Clinical Sciences, “G. d'Annunzio” University, Chieti, Italy; c Department of Innovative Technologies in Medicine & Odontoiatry, “G. d'Annunzio” University, Chieti, Italy

**Keywords:** Lung biopsy, Air embolism, Cerebral ischemia

## Abstract

Lung biopsy is an important interventional radiology procedure allowing the characterization of lesions with suspected malignancy. The most frequent complications are pneumothorax and hemorrhage. Air embolism is a rare but potentially fatal occurrence. In this case report, we present an air embolism after core needle CT-guided biopsy showing CT and MRI features that radiologists should expect in the everyday clinical practice.

## Introduction

Lung biopsy is an important interventional radiology procedure allowing the characterization of lesions with suspected malignancy. Core needle biopsy (CNB) and fine-needle aspiration (FNA) provide respectively a histologic or cytologic confirmation of malignancy. Compared to FNA, which allows only a cytological evaluation, CNB makes it possibile an histopathological diagnosis. A weakness of CNB is the higher complication rate compared to FNA [1]. The main biopsy approaches include bronchoscopic, CT-guided, and ultrasound-guided percutaneous needle biopsy. While the bronchoscopic approach is usually used for central lesions, CT-guided biopsy is the best approach for peripheral lesions that cannot be reached endoscopically [2].

The sample accuracy of US-guided biopsy and CT-guided biopsy is comparable [3]. In case of pleural or peripheral lung lesions, US guidance allows a significant reduction in procedure time, postprocedural pneumothorax and avoids ionizing radiation exposure [3]. Furthermore, ultrasound can be used for real-time visualization and multiplanar monitoring for blood vessels and accurate localization of target lesions that move during respiration [4].

The most frequent complications of CT-guided CNB include pneumothorax (25.3%) and bleeding (18%) which are generally easy to manage [5]. Several factors can increase procedural risks, such as the presence of emphysema or air in the biopsy target in case of cystic lesions. Gas embolism represents a rare complication occurring in less than 1% of cases. More in detail, there is only a 0.04%-0.07% risk of this gas embolism and one-third of cases led to death or sequelae [5,6]. These data may be underestimated given the scarce or absent clinical symptoms that can occur in cases of microembolisms [6]. The presence of underlying diseases such as cancer, COPD, arterial hypertension, diabetes, idiopathic pulmonary fibrosis, and asthma were more likely associated with symptoms [6]. Among symptomatic cases, 79.7% of postbiopsy gas embolism shows symptoms or signs immediately after or during the procedure. The most common symptom is loss of consciousness (42.9%) followed by cardiac (32.7%) and neurological symptoms (24.5%) [6]. In this case report we present an air embolism after CT-guided biopsy showing CT and MRI features that radiologists should know in their everyday clinical practice.

## Case report

**Fig. 1:**
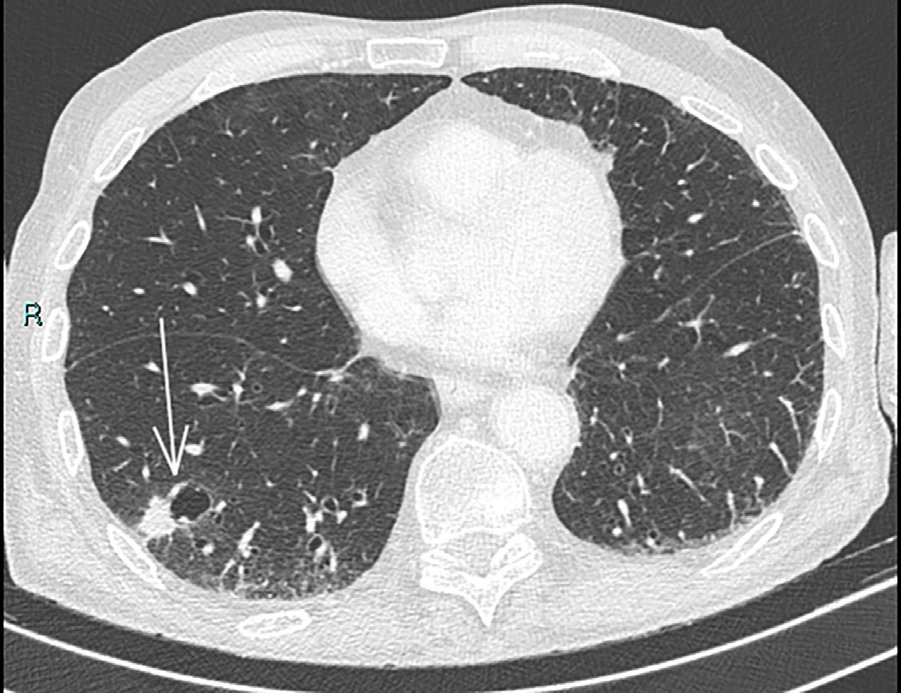
CT showing solid tissue with spiculated edges adjacent to a cystic formation (white arrow).

**Fig. 2:**
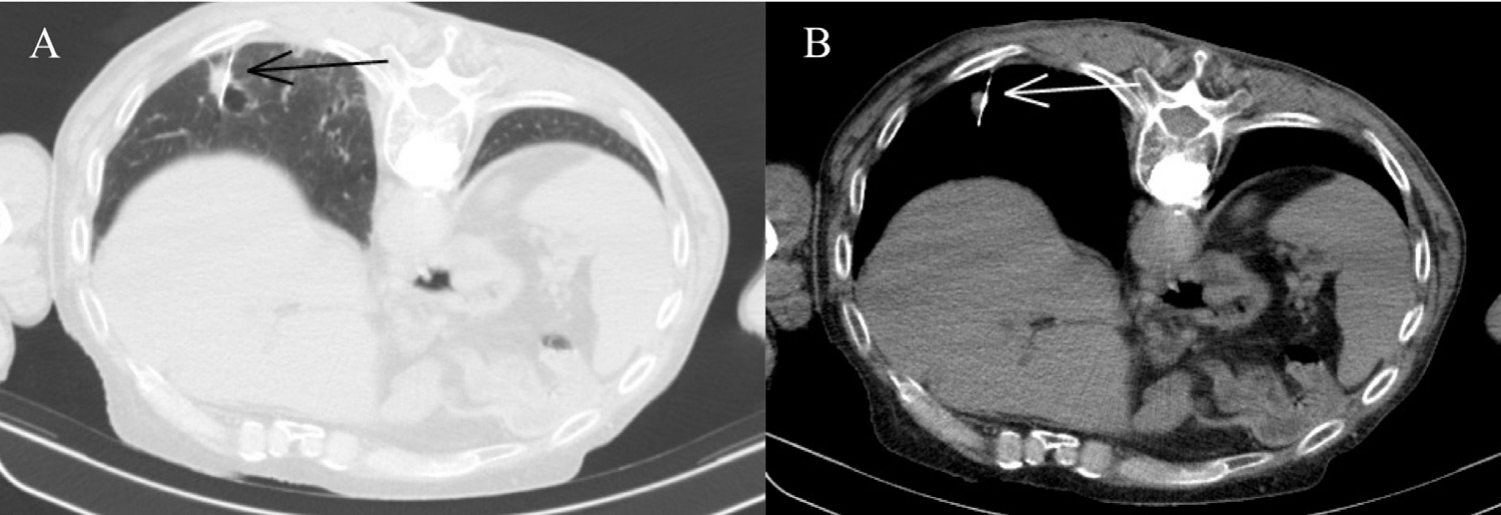
CT showing the percutaneous posterior intercostal access with the patient in prone position. Black (A) and white (B) arrows indicate the needle in the target lesion.

**Fig. 3:**
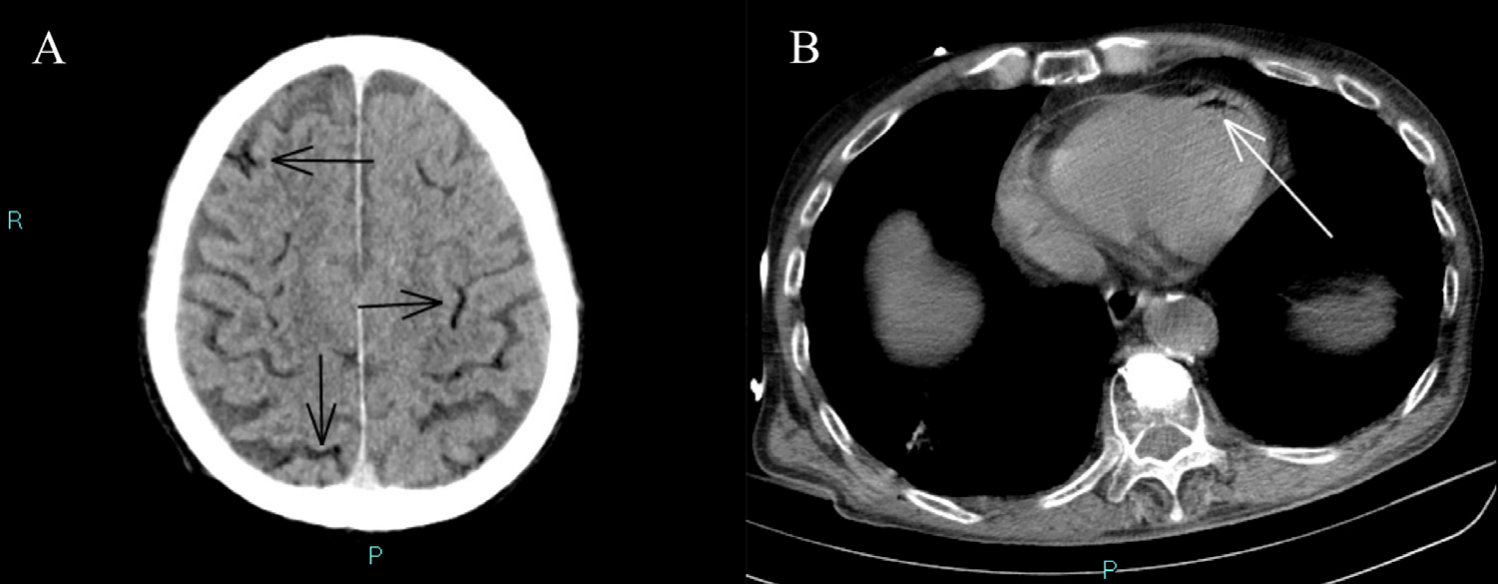
CT showing the presence of air microbubbles along the subarachnoid spaces of the bihemispheric convexity (black arrows in A) and in endocardial profile of the left ventricle (white arrow in B).

**Fig. 4:**
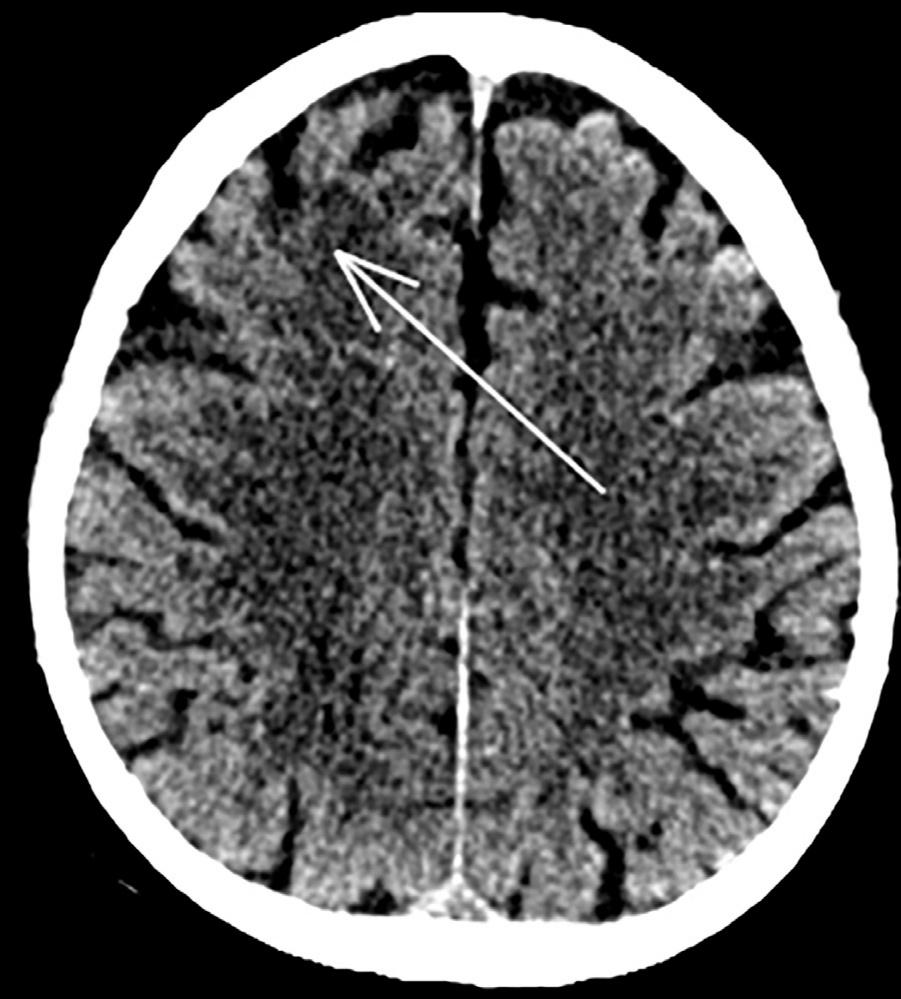
CT showing a small hypodensity (white arrow) in the right upper frontal cortico-subcortical region due to ischemic lesion.

**Fig. 5:**
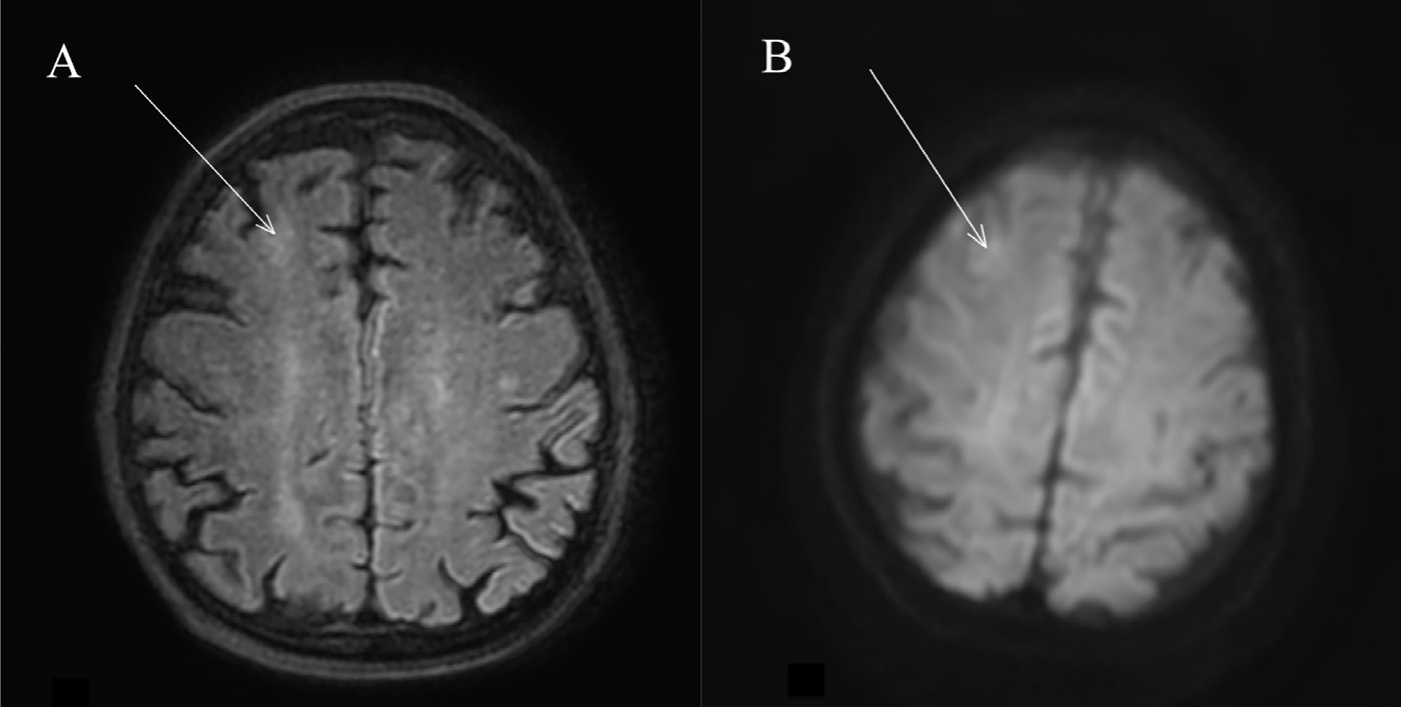
Fluid attenuated inversion recovery (FLAIR) and diffusion-weighted-imaging (DWI) in (A) and in (B). Areas of vague hyperintense signal alteration (white arrows in A and B) due to sub-acute ischemic lesions.

A 75 -year old man underwent lung CT at our Department for persistent cough. His medical history included smoking (20 cigarettes/day), left hemicolectomy for adenocarcinoma, and lumbar spine stabilization due to vertebral collapse induced by osteoporosis. The CT examination revealed the presence of solid tissue with spiculated edges adjacent to a cystic formation of 13 × 15 mm (on axial plane) located in the right lower lobe (Fig. 1). Given the suspicion for malignancy, it was decided to further characterize the lung lesion by CT-guided biopsy. A 18G x 100 mm coaxial needle was used for the procedure. It was decided to proceed with a CT-guided biopsy because the presence of the airborne component of the lesion would have potentially caused an acoustic barrier in ultrasound. The patient was moved to the prone position and a right dorsal percutaneous intercostal access was obtained (Fig. 2). Before starting the procedure, a local anesthesia using 2% lidocaine, was given. At the end of the biopsy procedure, the patient was moved to the supine position and immediately developed illness with hemoptysis and loss of consciousness. Arterial blood gas test and arterial pressure were normal. A few minutes after the illness, a brain and chest CT showed the presence of air microbubbles along the subarachnoid spaces of the bihemispheric convexity and along the endocardial profile of the left ventricle (Fig. 3). Alveolar hemorrhage in the basal pyramid of the right lower lobe involving in the site of biopsy was also demonstrated. The patient was admitted to intensive care unit and the 24-hours CT control showed a small hypodensity in the right upper frontal cortico-subcortical site, corresponding to a recent ischemic lesion (Fig. 4). The air density images previously reported in the subarachnoid space and left ventricle were no longer appreciable. In the following days, MRI was performed 18 days after the biopsy and confirmed the presence of hyperintense signal alteration in the frontal cortico-subcortical and right parietal areas. They did not show restricted diffusivity and were compatible with ischemic lesions in evolutive phase (Fig. 5).

## Discussion

Noncontrast CT scan is a highly sensitive imaging technique to detect the presence of small amounts of air [7]. Patients with suspected cerebral air embolism should undergo CT scan within the first hour from the onset, owing to rapid resorption of air by brain arterioles [7]. Air emboli shows a gyriform pattern consisting of small round or curvilinear hypodensities representing air in the cerebral cortical vessels [8]. It should be emphasised that late examination may not show the presence of air and this fact might explain the under-recognition of this disease [7]. The CT re-evaluations in the following hours/days show, as in our case, the resolution of the air embolic component with the progressive appearance of the classic hypodense ischemic areas [7].

As reported in the literature, there are several risk factors increasing the likelihood of gas embolism after CT-guided lung biopsy. The prone position of the patient, the location of the lesion in the lower lobes, the execution of endotracheal anesthesia, the localization of the lesion on a higher plane than the left atrium, the high number of biopsy samples, the presence of healthy and aerated lung tissue in the biopsy sample are statistically significant risk factors for air embolism [9]. Endotracheal anesthesia is a risk factor due to positive intra-alveolar or intra-bronchial pressure: high pressure increases the likelihood that air enters the vascular system [9]. If the lesion is located above the left atrium, pulmonary venous pressure is lower and therefore the likelihood of air rush into the vasculature increases in case of vessel damage. This explains the higher risk in the prone position: in this position, in fact, a lesion located in the lower lobes is located above the level of the atrium [9]. Furthermore, if the tip of the needle enters directly in the tumour, the risk of a gaseous embolism is lower because there is no interposition of aerated parenchyma [9]. A large number of biopsy samples increases the risk of air embolism because it increases the probability of puncturing and involving vascular and air structures creating fistulas [9].

In this case report, we hypothesized that, during the procedure, a communication between the air component of the pulmonary cystic lesion and a venous vessel was created. This hypothesis was supported by the fact that, at the end of the procedure, the cystic component of the lesion was collapsed, although it was not involved by the needle puncture. In this way, the air would have reached the left ventricle and from here, when passing from the prone to the supine position, it would have reached the brain causing gas embolism. At the same time, a communication between the airways and a pulmonary venous vessel occurred during the biopsy can't be excluded.

Some precautions may be adoped by radiologists to reduce the risk of air embolism during lung biopsy. In fact, the postbiopsy CT scan usually includes only the target area since it has the role to exclude pneumothorax and haemorrhage in the biopsy tract. However, the early detection of air embolism in the left atrium or ventricle can prevent air embolism in the systemic circulation. For this reason, a CT scan of the whole cardiac region with the patient in unchanged position respect to the biopsy procedure should be recommended to find any evidence of air embolism. If the CT images do not show air in the aforementioned areas, the patient can safely be placed back in the supine position. On the other hand, if the CT images show air in the left atrium or ventricle, it would be advisable to place the patient in the Trendelenburg position thus moving the air by gravity into the lower limbs and waiting its reabsorption [10,11].

Several cases described air embolism after lung biopsy [5,[12], [13], [14], [15], [16], [17], [18], [19], [20], [21], [22], [23], [24], [25], [26], [27]]. However, most of these studies included solid lung lesions with no air component. To the best of our knowledge, only 2 cases of solid lesions with airborne component were reported in literature in the last 10 years [11,17].

The management of patients with air embolism involves different types of intervention. Patients with cardiac arrest are treated with cardiopulmonary resuscitation, intubation, and in some cases admission to an intensive care unit [10]. The supportive treatment by administration of high concentration oxygen (100%) and the expansion of the intravascular volume is crucial to prevent further entry of air into the circulation. Hyperbaric oxygen therapy may be considered for more severe cases [28].

## Conclusion

Air embolism is a rare complication of lung biopsy procedures. Cystic lesions, such as the 1 discussed in this case report expose to a greater risk of gas embolism. The knowledge of CT and MRI radiological findings may helps radiologists to not underestimate this potentially fatal complication.

## Patient consent

Informed consent was obtained all the data referred to the patient were anonymized.

## Author contributions

All the authors were involved in patient management, wrote the report and consent to publication.
